# Comparison of Obesity-Related Indicators for Nonalcoholic Fatty Liver Disease Diagnosed by Transient Elastography

**DOI:** 10.5152/tjg.2023.23101

**Published:** 2023-10-01

**Authors:** Xinyi Tian, Ning Ding, Yingjie Su, Jiao Qin

**Affiliations:** 1 Department of Nephrology, The Affiliated Changsha Central Hospital, University of South China Hengyang Medical School, Changsha, Hunan, China; 2 Department of Emergency Medicine, The Affiliated Changsha Central Hospital, University of South China Hengyang Medical School, Changsha, Hunan, China

**Keywords:** Nonalcoholic fatty liver disease, obesity-related index, transient elastography, body roundness index, abdominal volume index

## Abstract

**Background/Aims:**

This study aimed to assess obesity-related indices in predicting nonalcoholic fatty liver disease (NAFLD) in the United States. These indices were analyzed separately in previous studies, but evidence comparing them together was still lacking.

**Materials and Methods:**

We analyzed data from 8126 individuals in the National Health and Nutrition Examination Survey (NHANES) database and measured their body mass index (BMI), body roundness index (BRI), a body shape index, conicity index, body adiposity index, abdominal volume index (AVI), and waist–hip ratio. We used logistic analyses with odds ratios to evaluate the association between obesity-related indices and NAFLD and compared their diagnostic ability by receiver operating characteristic (ROC) curves, areas under the curve (AUCs), and net reclassification improvement (NRI).

**Results:**

The AVI had the highest AUC (0.835 at controlled attenuation parameter [CAP] scores 263 dB/m and 0.831 at CAP scores 285 dB/m) in the ROC curve analysis. The AVI also showed better discriminatory ability than BMI (NRI = 0.0331 at CAP scores 263 dB/m and 0.0328 at CAP scores 285 dB/m), the same as BRI (NRI = 0.0283 at CAP scores 263 dB/m and 0.0272 at CAP scores 285 dB/m). In males, AVI (AUC = 0.8501 at CAP scores 263 dB/m and 0.8466 at CAP scores 285 dB/m) and BRI (AUC = 0.8517 at CAP scores 263 dB/m and 0.8497 at CAP scores 285 dB/m) had better predictive ability than BMI and similar to females. This was consistent across different age and race groups.

**Conclusion:**

AVI and BRI were better predictors of NAFLD than BMI.

**Main Points**

- Previous studies have analyzed obesity-related indicators separately, and there is a lack of evidence comparing them together. Several obesity-related indicators for nonalcoholic fatty liver disease diagnosed by transient elastography were compared in this research.
- For the population as a whole, abdominal volume index (AVI) had the greatest predictive ability.
- The distinguishing capacity of both body roundness index and AVI was better than body mass index.

## Introduction

Nonalcoholic fatty liver disease (NAFLD) was becoming increasingly widespread as the prevalence of diabetes and obesity increased, and it was the main cause of liver disease globally. The majority of hepatocellular carcinoma was associated with hepatitis virus infection, but NAFLD was becoming a major cause of hepatocellular carcinoma (HCC) in the United States. NAFLD had caused huge health and economic burden to patients, their families, and society.^1^ Liver biopsy was the gold standard for diagnosing NAFLD. However, biopsy was an invasive method that might lead to complications such as death and bleeding. There were also reports of sampling errors in NAFLD patients, which might affect the diagnosis and staging of the disease.^2^ As a result, noninvasive approaches for diagnosing NAFLD were urgently needed.^3^

The National Health and Nutrition Examination Survey (NHANES) database collected a large number of transient elastography (TE) examination data among the entire U.S. population in 2017-2020. Because it is non-invasive, simple, fast, easy to operate, repeatable, safe, and well tolerated, it could help doctors better assess the severity of liver fibrosis and steatosis, partially replace liver puncture biopsy, and reduce the need for clinical liver puncture. Transient elastography had been recommended by the American Academy of Hepatology (AASLD), the European Society of Hepatology (EASL), and the treatment of chronic hepatitis B as a significant method of clinical assessment of hepatitis C and B virus-related hepatic fibrosis. The index of the controlled attenuated parameter (CAP), which was a numerical evaluation, had been proved to have a good correlation between TE and hepatic steatosis, and it was superior to abdominal ultrasound;^4-6^ however, there was a lack of an appropriate threshold for the diagnosis of NAFLD. Studies had shown that the 263 and 285 thresholds were reliable, and more than 90% were sensitive for the diagnosis of NAFLD.^7^ At present, many articles had been published by using this critical value.^8,9^

NAFLD was a disease related to obesity. Obesity-related indicators could reflect diseases related to metabolism and health in the body. In recent years, many scholars had derived various obesity-related indexes such as weight, height, hip circumference, and waist circumference, based on different algorithms, such as body roundness index (BRI), conicity index (CI), abdominal volume index (AVI), waist-hip ratio (WHR), and a body shape index (ABSI) and had studied their correlation in hypertension, diabetes, heart failure, renal insufficiency, and so on.^10^ Previous studies had shown a correlation between WHR, BRI, AVI, and NAFLD.^11-13^ There was a literature review on NAFLD diagnosis using abdominal ultrasound to assess the diagnostic ability of various obesity indices and NAFLD and to evaluate which obesity index has a better ability to diagnose NAFLD.^14^ Based on the above, this study aimed to use the relevant data of TE in the NHANES database to compare various obesity-related indicators to diagnose NAFLD better.

## Materials and Methods

### Subjects and Study Design

**Figure 1.**
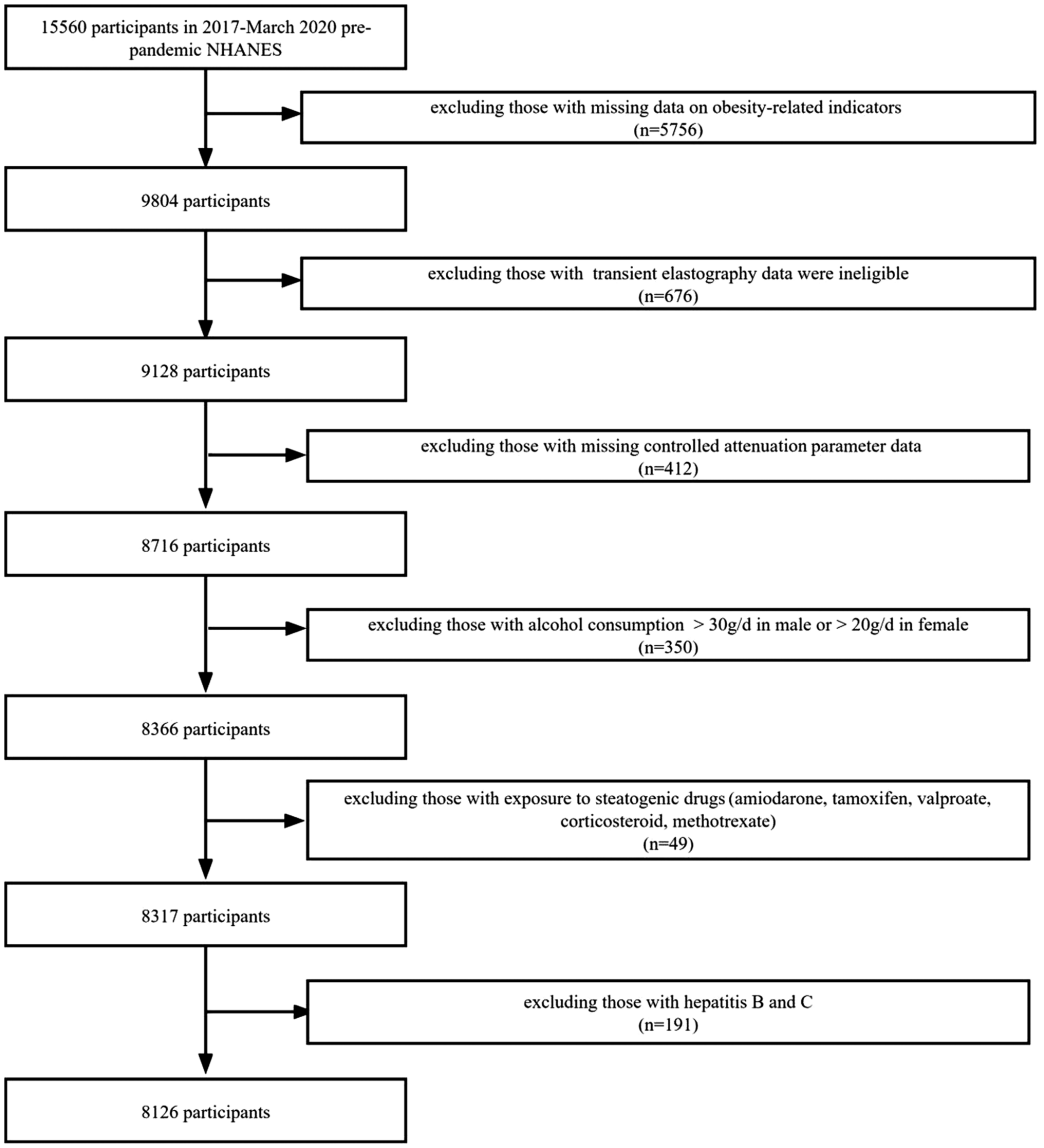
Flowchart of the study design and participants excluded from the study.

The most recent NHANES 2017-2020 data were examined in this study. To gather representative adults in the United States, the NHANES data use a multistaged, stratified, and clustered probability sample approach. Regarding inclusion and exclusion criteria, we referred to a previously described method.^8^ Of 15 560 participants in 2017, based on March 2020 pre-pandemic NHANES database, we excluded 5756 participants with missing data on obesity-related indicators and excluded 676 participants with TE data were ineligible. Next, we excluded 412 participants with missing controlled attenuation parameter data. We also excluded 191 participants with hepatitis B virus and hepatitis C virus, 350 participants with alcohol consumption (>30 g/day in men and >20 g/day in women), and 49 participants with exposure to steatogenic drugs (valproate, tamoxifen, methotrexate, corticosteroid, and amiodarone). A total of 8126 participants were included in the final cohort, all of whom had complete data (Figure 1).

### Ethics Approval and Consent to Participate

All methods in our research were performed in accordance with the Declaration of Helsinki. This study protocol was reviewed and approved by National Center for Health Statistics (NCHS) Ethics Review Board (approval number Protocol #2018-01). Written informed consent from all adult patients to participate in the study were obtained. Written informed consent from parents/guardian/next of kin for all vulnerable participants were obtained.

### Data Collection

We used TE to diagnose NAFLD. CAP was used by using TE (Fibroscan ®; Echosense) to detect and quantify liver steatosis. We adopt 2 CAP thresholds of 263 and 285, the sensitivity was more than 90%.^7^ This was done using only the M probe, as the CAP algorithm was unique to the device. Each patient was successfully measured 10 times, and this study only considered cases that were successfully collected 10 times. Therefore, checks with less than 10 successful measurements were considered as failures. 

The following equations were used to calculate the obesity-related indices AVI, ABSI, BRI, body mass index (BMI), body adiposity index (BAI), CI, and WHR.^15-19^



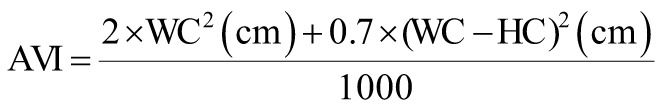





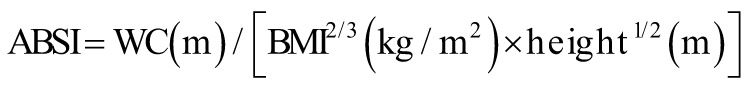





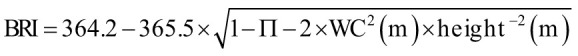





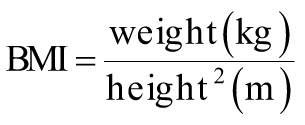





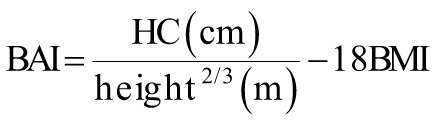





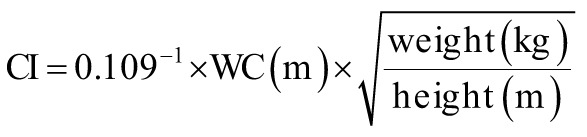





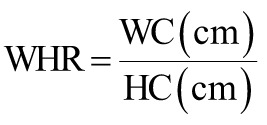



where AVI is the abdominal volume index; ABSI is a body shape index; BMI is the body mass index; BRI is the body roundness index; BAI is the body adiposity index; CI is the conicity index; HC is the hip circumference; WHR is the waist–hip ratio; and WC is the waist circumference.

### Statistical Analysis

The survey-weighted mean were applied to represent continuous variables, including BMI, BRI, ABSI, CI, BAI, AVI, and WHR. The *P*-value was by survey-weighted linear regression. The survey-weighted percentage were utilized to represent categorical variables, including age, gender, and race. The *P*-value was by survey-weighted chi-square test. All subjects were classified as NAFLD and non-NAFLD based on different cutoffs of CAP scores (263 dB/m and 285 dB/m). The odds ratios (ORs) and 95% CIs of these obesity-related indicators with NAFLD were determined using weighted logistic analyses. By creating receiver operating characteristic (ROC) curves, area under the curve (AUC), and net reclassification improvement (NRI), we compared the diagnostic ability of obesity-related indices to diagnose NAFLD. The data was analyzed using the statistical software packages R (http://www.r-project.org) and EmpowerStats (http://www. empowerstats.com, X&Y Solutions, Inc., Boston, MA, USA). It was deemed statistically significant when the* P*value was <.05.

## Results

### Characteristics of the Study Population

This study included a total of 8126 participants. Among these subjects, with CAP scores (263 dB/m) as the diagnostic standard, 3617 persons had NAFLD. Taking CAP scores (285 dB/m) as the diagnostic standard, 2632 persons had NAFLD. 

Table 1 showed the characteristics of the whole study population based on different CAP score cutoffs. Males were shown to have a greater proportion of NAFLD than females. NAFLD had the highest prevalence among people ≥60 years of age. Non-Hispanic White made up the majority of NAFLD patients. Subjects with NAFLD had significantly greater BMI, BRI, ABSI, CI, BAI, AVI, and WHR than those without NAFLD (all *P* < .0001).

Odds ratio for NAFLD based on different CAP scores risk across quartiles of each index.

Table 2 demonstrated that the obesity-related indices studied were significantly associated with NAFLD (*P* < .0001). The ORs for NAFLD based on CAP scores 263 dB/m or CAP scores 285 dB/m both increased across the quartiles of each index.

### Receiver Operating Characteristic Curves and Area Under the Curve for Indices in Identifying Nonalcoholic Fatty Liver Disease

**Figure 2.**
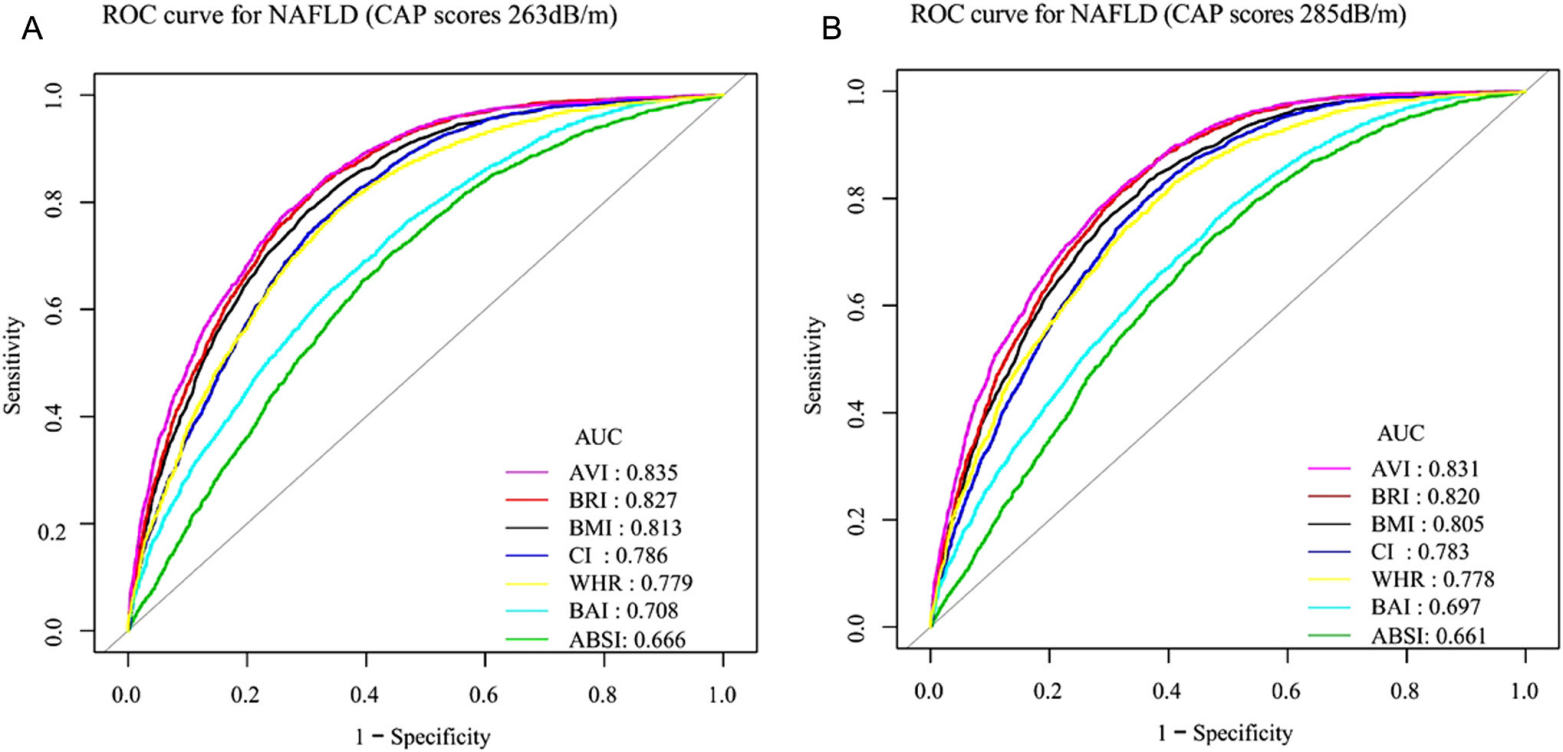
Comparison of the predictive value of obesity-related indicators for the diagnosis of NAFLD based on CAP scores (263 dB/m) and CAP scores (285 dB/m). CAP, controlled attenuation parameter; NAFLD, nonalcoholic fatty liver disease.

Table 3 illuminated that BRI, BMI, and AVI were among the top 3 predictive indices, the AUCs of these indices were over 0.8. AVI had the greatest AUC of 0.8353 (CAP scores 263 dB/m) or 0.8305 (CAP scores 285 dB/m). As shown in Figure 2A, AVI had the greatest predictive ability (AUC = 0.835), and BRI had the next best diagnostic ability (AUC = 0.827), followed by BMI (AUC = 0.813). In Figure 2B, AVI also exhibited the best predictive capacity (AUC = 0.831), followed by BRI (AUC = 0.820) and BMI (AUC = 0.805). Table 4 showed that comparing BMI, BRI, and AVI when using CAP scores 263 dB/m as the critical value to diagnose NAFLD, the distinguishing capacity of both BRI and AVI was better than BMI and the NRI was 0.0283 (*P* = .0002) and 0.0331 (*P*< .0001), respectively. While using CAP scores 285 dB/m as the critical value, BRI and AVI both showed better distinguishing capacity than BMI and the NRI was 0.0272 (*P *= .0008) and 0.0328 (*P* < .0001), respectively.

### Comparison of Obesity-related Indices in Different Subgroups for the Diagnosis of Nonalcoholic Fatty Liver Disease

**Figure 3.**
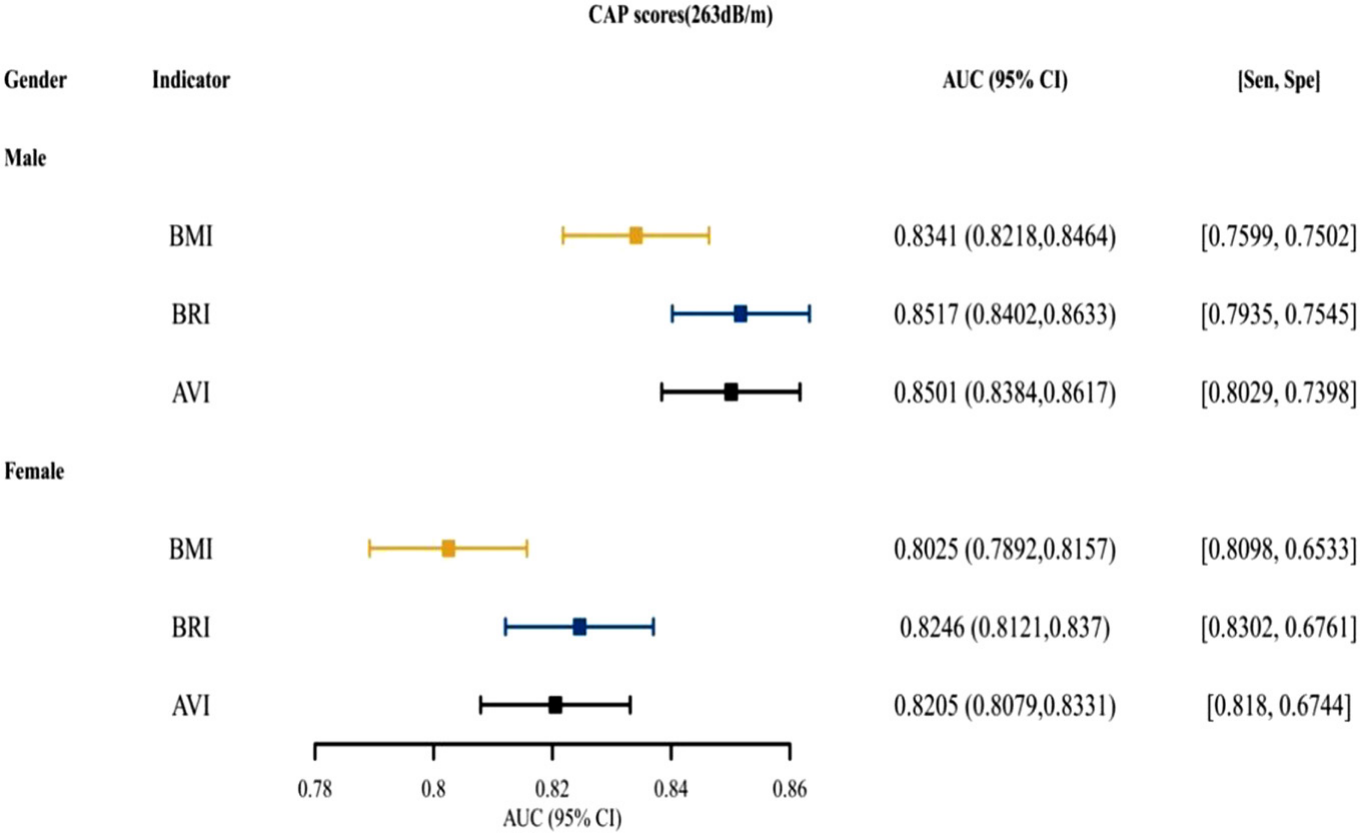
The AUC, specificity, and sensitivity of BMI, BRI, and AVI for predicting NAFLD (CAP scores 263 dB/m) in different gender. AUC, area under the curve; AVI, abdominal volume index; BMI, body mass index; BRI, body roundness index; CAP, controlled attenuation parameter; NAFLD, nonalcoholic fatty liver disease.

**Figure 4.**
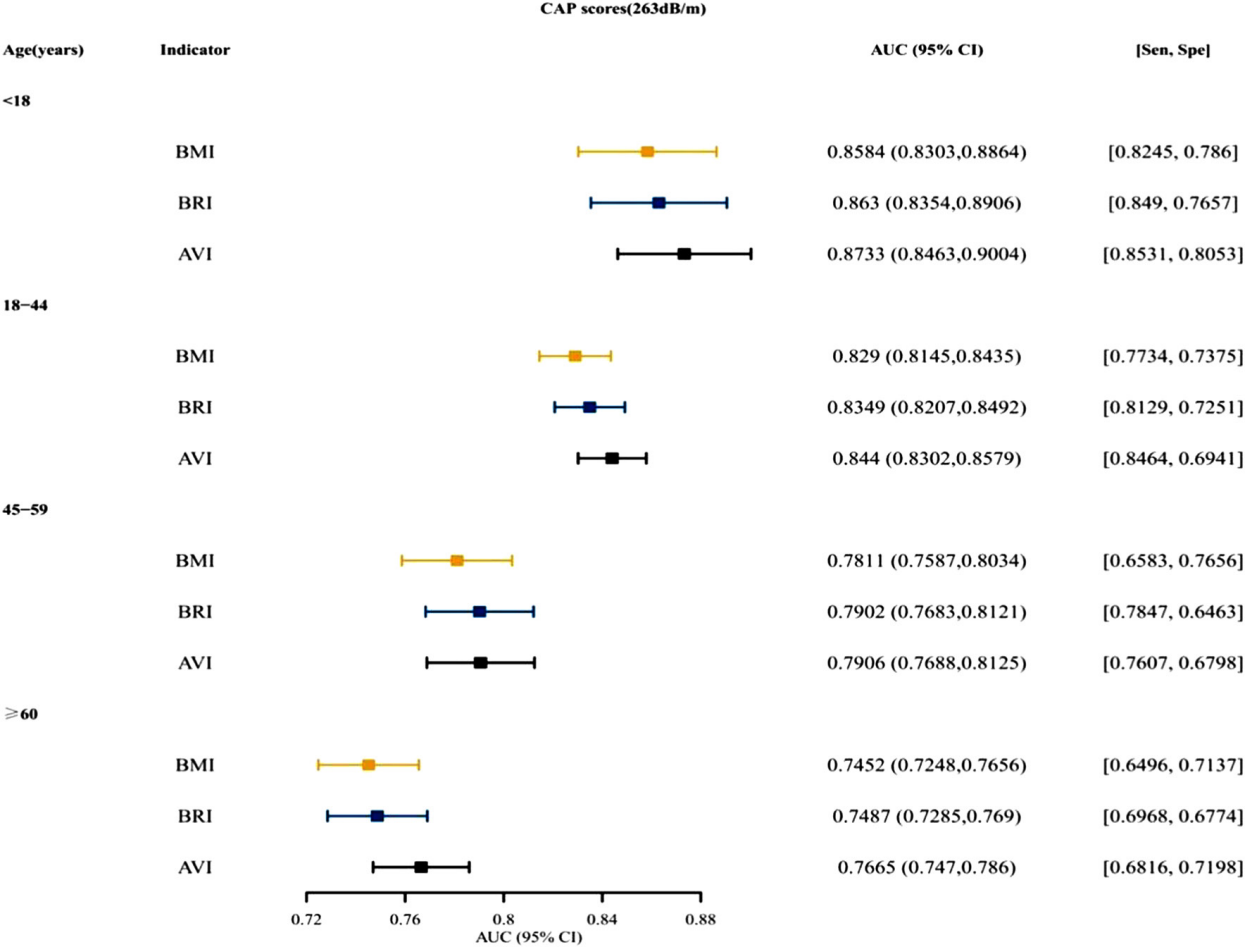
The AUC, specificity, and sensitivity of BMI, BRI, and AVI for predicting NAFLD (CAP scores 263 dB/m) in different ages. AUC, area under the curve; AVI, abdominal volume index; BMI, body mass index; BRI, body roundness index; CAP, controlled attenuation parameter; NAFLD, nonalcoholic fatty liver disease.

**Figure 5.**
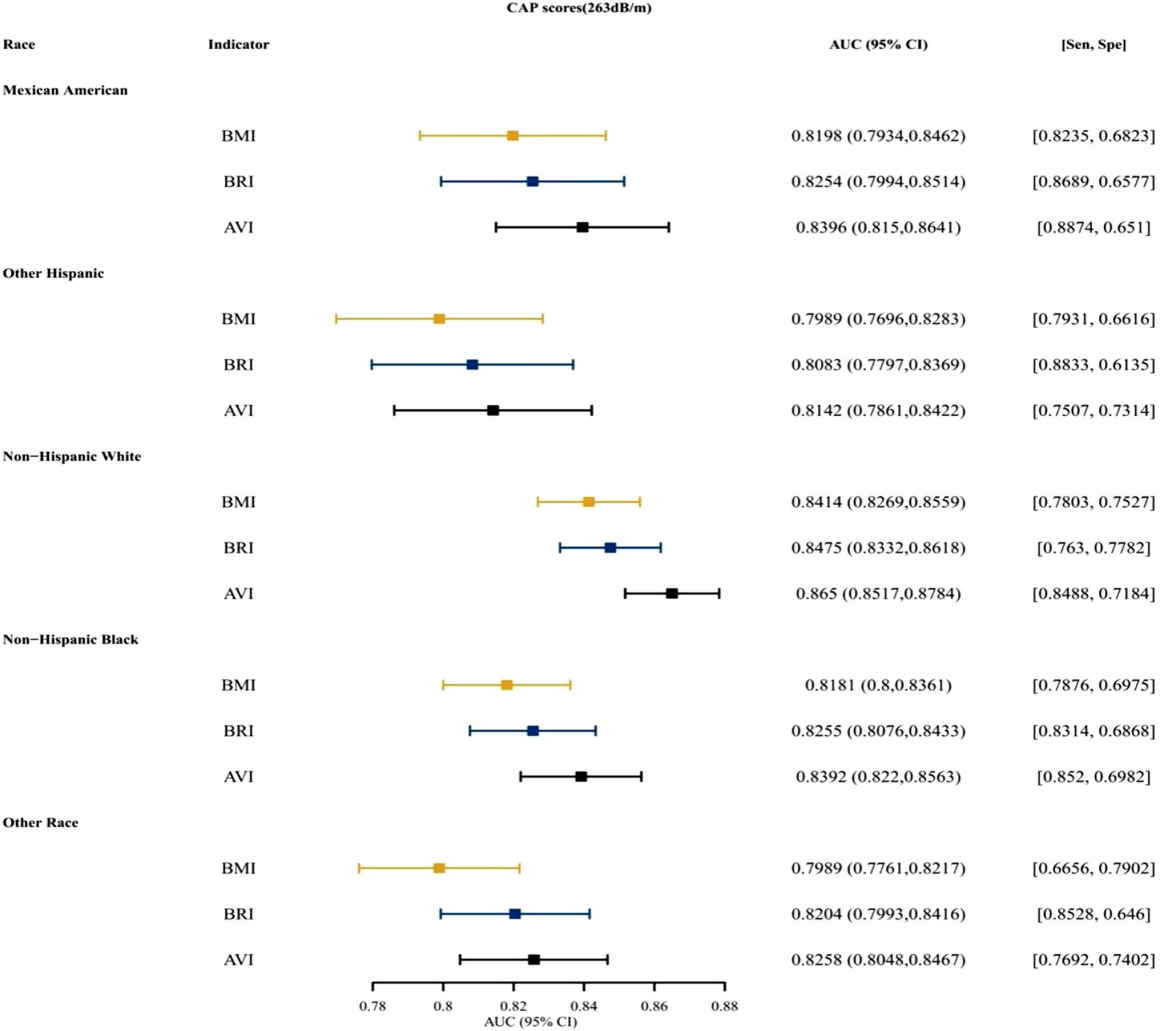
The AUC, specificity, and sensitivity of BMI, BRI, and AVI for predicting NAFLD (CAP scores 263 dB/m) in different races. AUC, area under the curve; AVI, abdominal volume index; BMI, body mass index; BRI, body roundness index; CAP, controlled attenuation parameter; NAFLD, nonalcoholic fatty liver disease.

**Supplementary Figure 1.**
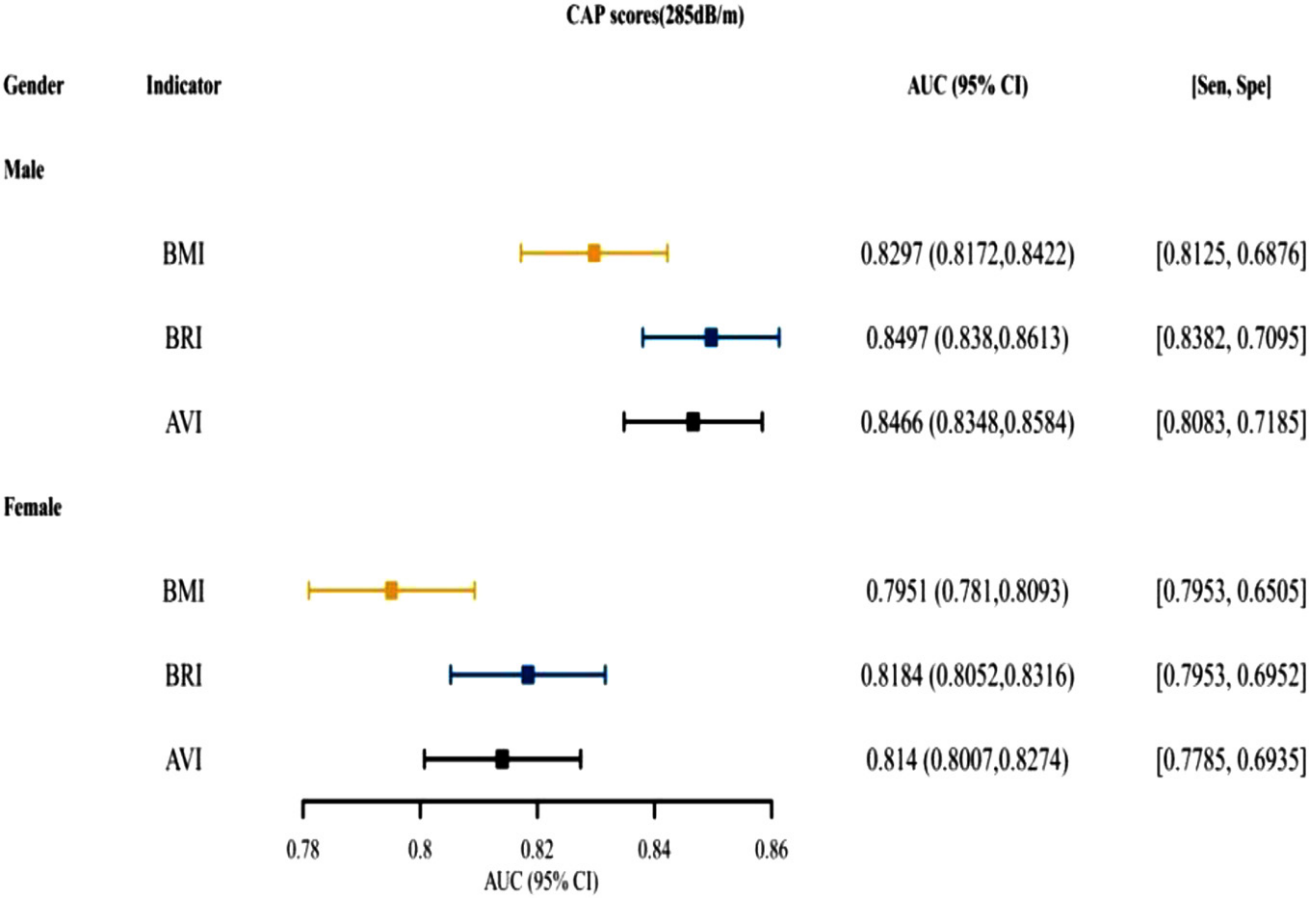
The AUC, specificity, sensitivity of BMI, BRI, AVI for predicting NAFLD (CAP scores 285dB/m) in different gender. AUC, area under the curve; BMI, body mass index; BRI, body roundness index; AVI, abdominal volume index; NAFLD, nonalcoholic fatty liver disease; CAP, controlled attenuated parameter.

**Supplementary Figure 2.**
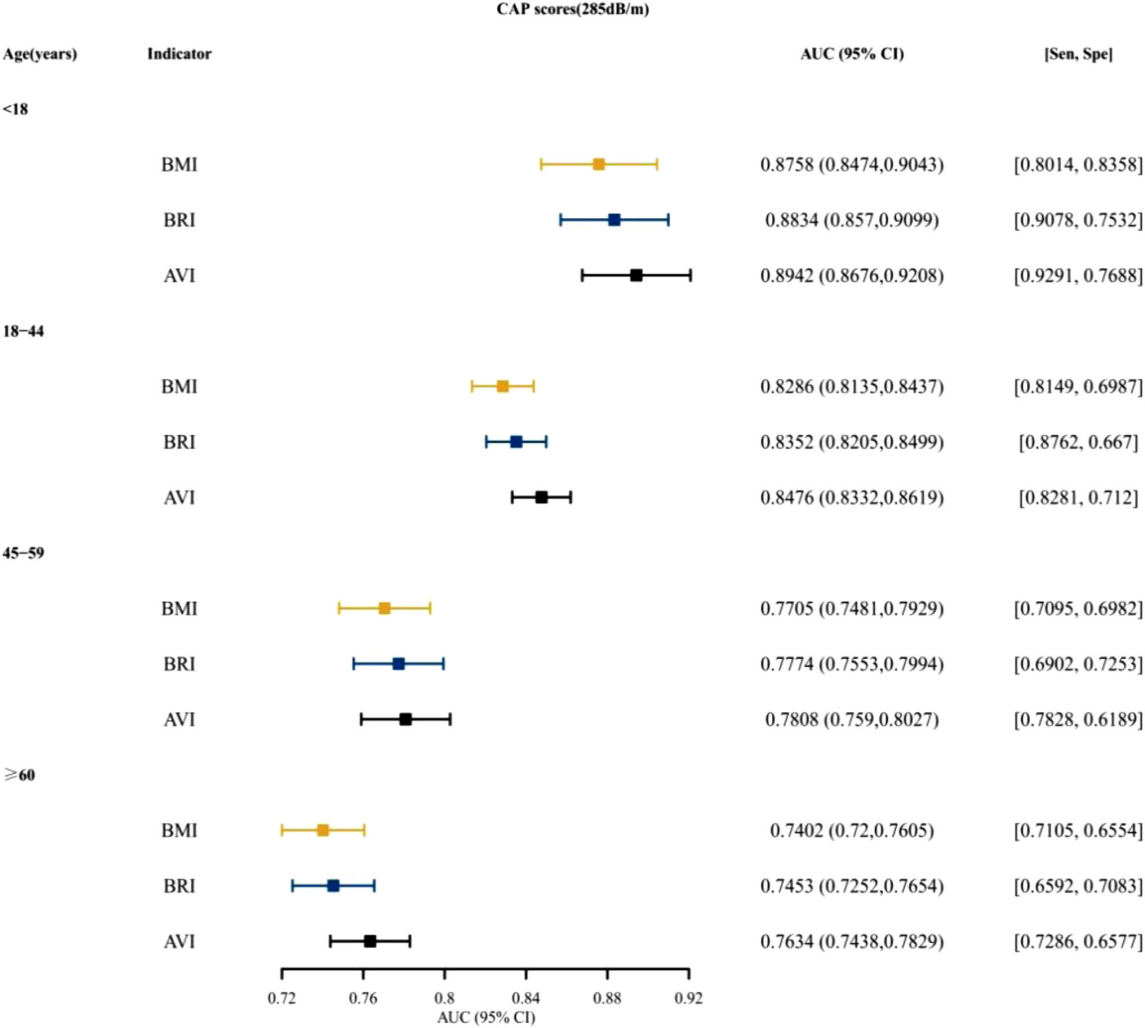
The AUC, specificity, sensitivity of BMI, BRI, AVI for predicting NAFLD (CAP scores 285dB/m) in different age. AUC, area under the curve; BMI, body mass index; BRI, body roundness index; AVI, abdominal volume index; NAFLD, nonalcoholic fatty liver disease; CAP, controlled attenuated parameter.

**Supplementary Figure 3.**
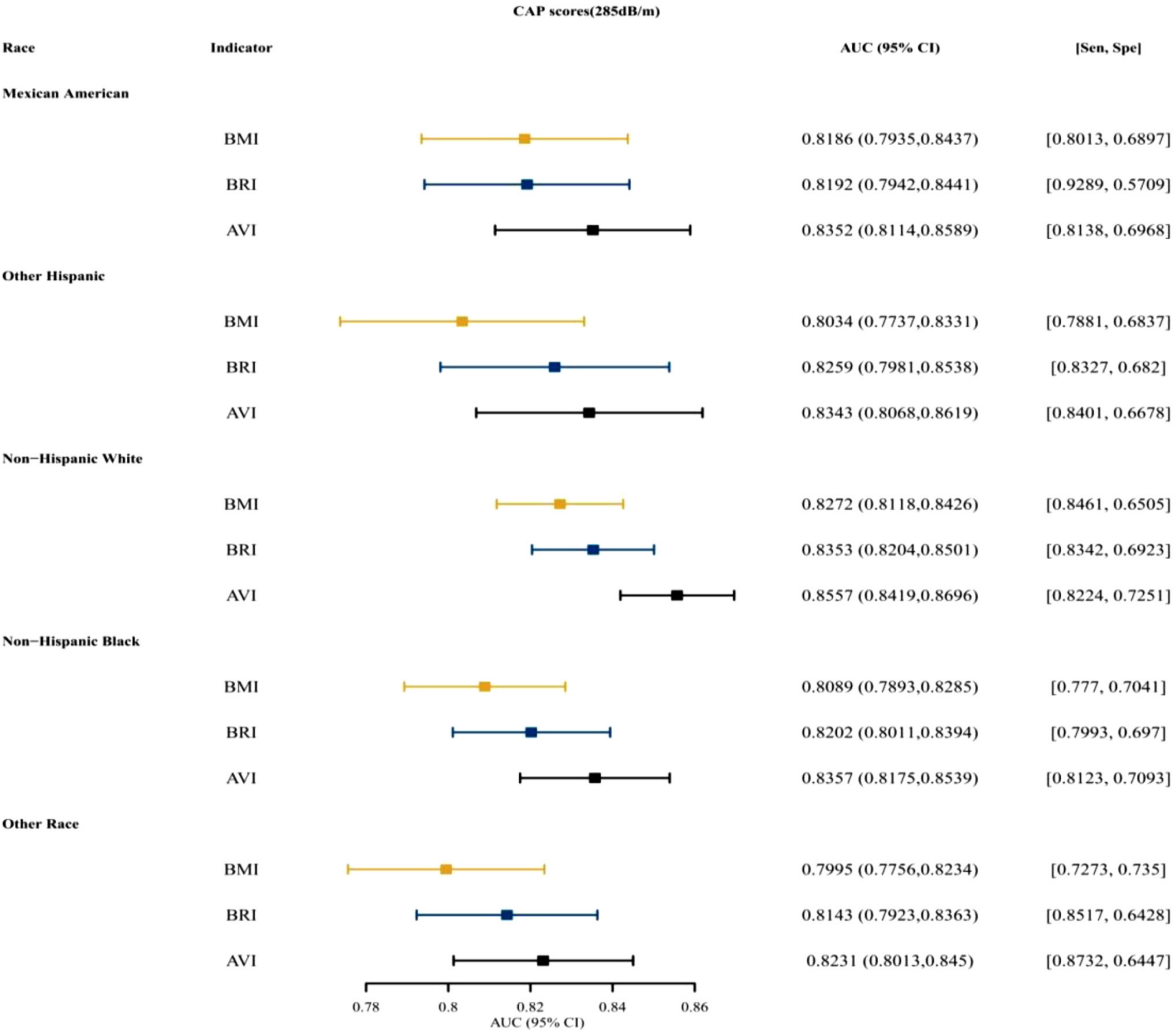
The AUC, specificity, sensitivity of BMI, BRI, AVI for predicting NAFLD (CAP scores 285 dB/m) in different race. AUC, area under the curve; BMI, body mass index; BRI, body roundness index; AVI, abdominal volume index; NAFLD, nonalcoholic fatty liver disease; CAP, controlled attenuated parameter.

Figure 3 and Supplementary Figure 1 presented that for both males and females, BRI was the top indices for predictive ability, followed by AVI. Figure 4 and Supplementary Figure 2 presented the AUCs of the indices for NAFLD in patients who were <18, 18~ 44, 45-59, and ≥60 years old. For different age groups, as indices for predictive ability, AVI and BRI were better than BMI. Figure 5 and Supplementary Figure 3 presented the AUCs of the indices for NAFLD in patients who were of different races, which showed that AVI and BRI were in the top 2 indices for predictive ability.

## Discussion

This cross-sectional study used the relevant data of TE in the NHANES database, and the diagnostic ability and cutoff value of obesity-related indices to diagnose NAFLD were thoroughly examined. And for predictive ability, we found that BRI and AVI were both better than the traditional obesity-related index BMI.

NAFLD was an obesity-related disease. With little or no alcohol consumption, NAFLD was defined as steatosis in more than 5% of hepatocytes. The pathological progress of NAFLD initially followed the process of “3 strikes”: lipotoxicity, inflammation, and, namely, steatosis. Inflammatory mediators, steatosis, and oxidative stress all played a part in the development of NAFLD.^20^ Obesity had been linked to an increase in the prevalence and severity of NAFLD in several studies: obesity was not only related to simple steatosis (SS) but also related to advanced diseases such as nonalcoholic steatohepatitis (NASH), NASH-related liver hardening and hepatocellular carcinoma.^21^

Obesity-related indicators could reflect diseases related to metabolism and health in the body. BMI, as a traditional indicator for defining obesity, had many drawbacks. It could not distinguish between muscle and fat, is inaccurate in predicting body fat percentage,^22^ and is not a good method for measuring the risk of heart attack, stroke, or death.^23^ BRI had been used by some researchers to predict body fat and visceral adipose tissue percentages and to preliminarily summarize a person’s physical health.^17^ In Peruvian adults, BRI had been discovered as a potentially valuable clinical predictor of metabolic syndrome.^24^ And there was a study that reported a high correlation between BRI and NAFLD.^12^ BRI could be used as a single suitable anthropometric measure in simultaneously identifying a cluster of cardiometabolic abnormalities (CMAs) compared to BMI.^25^ AVI was highly correlated with glucose metabolism dysfunction.^26^ AVI also had a low false-negative rate and a larger percentage of detected NAFLD, according to certain researchers.^13^ AVI has a higher diagnostic performance than BMI in the diagnosis of metabolic syndrome and a better correlation with metabolic diseases.^27^ A previous study had compared the ability of different obesity-related indices to diagnose NAFLD, but they were based on abdominal ultrasound diagnosis. They found that BMI has the largest AUC among all participant indicators, and considering the influence of gender, BMI and BRI have a high correlation with NAFLD and high diagnostic ability for NAFLD.^14^ This study found that using AVI and BRI as diagnostic predictors of NAFLD was better than BMI, and stratified diagnosis by gender, age, and race was still valid.

Compared with previous studies, this study was based on TE to diagnose NAFLD, with a large sample size. Transient elastography was a digital index and had an objective evaluation standard. This study had the following limitations: first, we used TE to diagnose NAFLD, while the gold standard was biopsy, which might have deviation. Second, data on other confounding factors, such as exercise, smoking, and drinking status, were not included in the analysis. Finally, because this was a cross-sectional investigation, it was impossible to prove causal links or long-term clinical effects.

## Conclusion

In this study, specific indicators for predicting NAFLD were found in American subjects with 263 and 285 CAP thresholds. The results of this study showed that BRI and AVI had better diagnostic ability for NAFLD than BMI. And when stratified diagnosis by sex, age, and race, this conclusion was still tenable.

## Figures and Tables

**Table 1.** Description of Subjects Based on Different Controlled Attenuation Parameter Score Cutoffs

Characteristics	Definition of NAFLD Based on Different CAP Score Cutoffs				*P*
	<263	≥263	<285	≥285	
n	4509	3617	5494	2632	
Age	38.84 (37.74, 39.95)	48.36 (47.02, 49.71)	40.39 (39.30, 41.47)	48.73 (47.33, 50.12)	<.0001
<18	15.65 (13.91, 17.57)	4.43 (3.72, 5.26)	14.06 (12.48, 15.80)	3.54 (2.79, 4.49)	
18-44	47.75 (44.52, 50.99)	36.85 (33.58, 40.26)	45.63 (42.85, 48.44)	37.13 (32.99, 41.47)	
45-59	17.16 (15.46, 19.00)	28.90 (26.54, 31.38)	19.43 (17.70, 21.29)	28.62 (25.03, 32.50)	
≥60	19.44 (16.98, 22.17)	29.82 (26.04, 33.89)	20.88 (18.31, 23.70)	30.71 (26.49, 35.27)	
Gender					<.0001
Male	44.89 (42.03, 47.78)	53.51 (50.81, 56.19)	45.49 (43.34, 47.67)	55.43 (51.87, 58.93)	
Female	55.11 (52.22, 57.97)	46.49 (43.81, 49.19)	54.51 (52.33, 56.66)	44.57 (41.07, 48.13)	
Race					<.0001
Mexican American	7.82 (5.83, 10.42)	12.11 (9.08, 15.98)	7.96 (5.93, 10.61)	13.38 (9.89, 17.87)	
Other Hispanic	8.33 (6.71, 10.29)	7.33 (5.79, 9.22)	8.19 (6.63, 10.06)	7.25 (5.53, 9.45)	
Non-Hispanic White	60.26 (54.55, 65.70)	61.47 (55.90, 66.76)	60.53 (55.06, 65.75)	61.36 (55.06, 67.30)	
Non-Hispanic Black	13.21 (10.14, 17.03)	9.10 (6.87, 11.98)	12.80 (9.80, 16.56)	8.43 (6.39, 11.04)	
Other race	10.39 (8.16, 13.14)	9.99 (7.96, 12.46)	10.51 (8.32, 13.21)	9.59 (7.51, 12.16)	
BMI	25.47 (25.14, 25.80)	33.02 (32.56, 33.48)	26.35 (26.05, 26.64)	34.01 (33.47, 34.55)	<.0001
BRI	4.09 (3.98, 4.20)	6.74 (6.57, 6.91)	4.39 (4.30, 4.49)	7.09 (6.89, 7.28)	<.0001
ABSI	0.08 (0.08, 0.08)	0.08 (0.08, 0.08)	0.08 (0.08, 0.08)	0.08 (0.08, 0.08)	<.0001
CI	1.25 (1.24, 1.25)	1.35 (1.34, 1.35)	1.26 (1.25, 1.26)	1.36 (1.35, 1.36)	<.0001
BAI	28.69 (28.38, 29.00)	34.17 (33.63, 34.72)	29.40 (29.13, 29.67)	34.75 (34.07, 35.43)	<.0001
AVI	16.15 (15.83, 16.47)	24.24 (23.75, 24.72)	17.04 (16.76, 17.33)	25.40 (24.85, 25.94)	<.0001
WHR	0.88 (0.88, 0.89)	0.96 (0.96, 0.97)	0.89 (0.89, 0.89)	0.97 (0.97, 0.98)	<.0001

Mean +/− SD for BMI, BRI, ABSI, CI, BAI, AVI, and WHR.* P*-value was calculated by Kruskal–Wallis rank sum test.

% for age, gender, and race. *P*-value was calculated by chi-square test.

ABSI, a body shape index; AVI, abdominal volume index; BAI, body adiposity index; BMI, body mass index; BRI, body roundness index; CAP, controlled ­attenuation parameter; CI, conicity index; NAFLD, nonalcoholic fatty liver disease; WHR, waist–hip ratio.

**Table 2.** Odds Ratio for Nonalcoholic Fatty Liver Disease Based on Different Controlled Attenuation Parameter Scores Stratified by Quartiles for Obesity-Related Indicators

	Q1	Q2 (OR, 95% CI, *P*)	Q3 (OR, 95% CI, *P*)	Q4 (OR, 95% CI, *P*)	*P*
CAP scores (263 dB/m)					
BMI	Ref.	5.63 (3.98, 7.97)	17.20 (12.63, 23.42)	50.11 (33.83, 74.23)	<.0001
BRI	Ref.	7.95 (5.85, 10.81)	23.18 (17.91, 30.01)	72.08 (48.66, 106.77)	<.0001
ABSI	Ref.	2.05 (1.67, 2.52)	3.84 (3.11, 4.74)	4.87 (3.88, 6.13)	<.0001
CI	Ref.	5.40 (4.10, 7.13)	15.82 (12.30, 20.37)	30.47 (23.08, 40.23)	<.0001
BAI	Ref.	2.57 (2.06, 3.19)	4.21 (3.17, 5.60)	8.34 (6.36, 10.94)	<.0001
AVI	Ref.	8.37 (6.01, 11.67)	28.50 (22.16, 36.64)	99.13 (69.36, 141.69)	<.0001
WHR	Ref.	3.85 (2.89, 5.14)	9.70 (7.23, 13.02)	24.40 (17.78, 33.48)	<.0001
CAP scores (285 dB/m)					
BMI	Ref.	6.65 (4.64, 9.53)	19.36 (12.99, 28.85)	59.38 (37.27, 94.62)	<.0001
BRI	Ref.	9.10 (6.72, 12.33)	29.65 (21.64, 40.62)	87.90 (57.53, 134.32)	<.0001
ABSI	Ref.	1.91 (1.48, 2.46)	3.53 (2.78, 4.49)	4.64 (3.44, 6.27)	<.0001
CI	Ref.	5.59 (3.77, 8.28)	16.16 (10.89, 23.96)	35.02 (24.39, 50.28)	<.0001
BAI	Ref.	2.97 (2.24, 3.95)	4.67 (3.57, 6.11)	8.31 (5.97, 11.57)	<.0001
AVI	Ref.	13.87 (9.72, 19.80)	41.41 (30.23, 56.75)	162.07 (115.38, 227.67)	<.0001
WHR	Ref.	3.89 (2.88, 5.26)	8.81 (6.10, 12.71)	24.26 (17.86, 32.95)	<.0001

ABSI, a body shape index; AVI, abdominal volume index; BAI, body adiposity index; BMI, body mass index; BRI, body roundness index; CAP, controlled ­attenuation parameter; CI, conicity index; NAFLD, nonalcoholic fatty liver disease; OR, odds ratio; WHR, waist–hip ratio.

**Table 3.** The Area Under the Curve, Best Threshold, Specificity, and Sensitivity of Obesity-Related Indices for Predicting Nonalcoholic Fatty Liver Disease Diagnosed by Different Controlled Attenuation Parameter Score Cutoffs

Indicator	ROC Area (AUC)	Best Threshold	Specificity	Sensitivity
CAP scores (263 dB/m)				
BMI	0.8126	27.4500	0.6953	0.7860
BRI	0.8268	4.6831	0.6780	0.8316
ABSI	0.6662	0.0796	0.5564	0.7064
CI	0.7863	1.2869	0.6762	0.7636
BAI	0.7079	28.6180	0.5602	0.7360
AVI	0.8353	18.5868	0.7336	0.7810
WHR	0.7788	0.9086	0.6463	0.7868
CAP scores (285 dB/m)				
BMI	0.8052	27.4500	0.6321	0.8343
BRI	0.8200	5.0530	0.6784	0.8153
ABSI	0.6611	0.0796	0.5220	0.7321
CI	0.7828	1.2930	0.6442	0.7926
BAI	0.6975	28.6238	0.5197	0.7618
AVI	0.8305	18.7857	0.6778	0.8214
WHR	0.7776	0.9087	0.5897	0.8294

ABSI, a body shape index; AUC, area under the curve; AVI, abdominal volume index; BAI, body adiposity index; BMI, body mass index; BRI, body roundness index; CAP, controlled attenuation parameter; CI, conicity index; ROC, receiver operating characteristic; WHR, waist–hip ratio.

**Table 4.** Comparison of Body Mass Index, Body Roundness Index, and Abdominal Volume Index in Discriminating Capacity of Nonalcoholic Fatty Liver Dsease Based on Different Controlled Attenuation Parameter Score Cutoffs

	BRI vs. BMI	AVI vs. BMI	
CAP scores (263 dB/m)			
RI for NAFLD	0.0456 (*P *< .0001)		−0.0053 (*P* = .3611)
RI for no NAFLD	−0.0173 (*P *= .0009)		0.0384 (*P* < .0001)
Net reclassification index	0.0283 (*P *= .0002)		0.0331 (*P* < .0001)
CAP scores (285 dB/m)			
RI for NAFLD	−0.0190 (*P* = .0034)		−0.0129 (*P* = .0435)
RI for no NAFLD	0.0462 (*P *< .0001)		0.0457 (*P* < .0001)
Net reclassification index	0.0272 (*P *= .0008)		0.0328 (*P* < .0001)

AVI, abdominal volume index; BMI, body mass index; BRI, body roundness index; CAP, controlled attenuation parameter; NAFLD, nonalcoholic fatty liver disease.
